# Effects of Shading-Induced Senescence and Retention of Lower-Canopy Needles on Photosynthetic Dynamics in Upper-Canopy Needles of *Cunninghamia lanceolata*

**DOI:** 10.3390/plants15192948

**Published:** 2026-09-27

**Authors:** Yueyue He, Yu Zhang, Qi Liu, Zhihao Li, Mengyuan Mao, Shubin Li, Lili Zhou

**Affiliations:** 1 College of Forestry, Fujian Agriculture and Forestry University, Fuzhou 350002, China; heeyy0210@126.com (Y.H.); 17805926186@163.com (Y.Z.); lllys021@126.com (Q.L.); fjlishubin@126.com (S.L.); 2 Chinese Fir Engineering Research Center of National Forestry and Grassland Administration, Fuzhou 350002, China; 3 College of Geography and Oceanography, Minjiang University, Fuzhou 350108, China; lzh880881@126.com (Z.L.); maooomy@163.com (M.M.); 4 College of Resources and Environment, Fujian Agriculture and Forestry University, Fuzhou 350002, China

**Keywords:** Chinese fir, light heterogeneity, marcescence, chlorophyll fluorescence, gas exchange, coupling and decoupling, antagonistic interactions

## Abstract

Spatial light heterogeneity within forest canopies drives organ-level competition and whole-plant functioning, yet how shade-induced lower-canopy senescence and retention affect upper-canopy photosynthesis in marcescent species remains unclear. In this study, three-year-old Chinese fir [*Cunninghamia lanceolata* (Lamb.) Hook.] seedlings were subjected to half-canopy (moderate shading, T1) and three-quarter canopy (severe shading, T2) treatments, alongside a non-shaded control (CK), to investigate the monthly and diurnal dynamics of intra-canopy photosynthetic and photochemical traits. The results showed that long-term shading significantly reduced lower-needle photosynthetic capacity and accelerated senescence, as limitations shifted from stomatal (during January–April) to non-stomatal (during May–June), ultimately impairing photosystem II (PSII) (indicated by a significant decline in *F*_v_/*F*_m_) and inducing needle retention. Concurrently, upper needles exhibited pronounced non-autonomous responses with the net photosynthetic rate (*P*_n_), stomatal conductance (*G*_s_), and water-use efficiency (WUE) decreasing with increasing shading intensity. Compared with the CK, the reduction in *P*_n_ reached 61.99% under severe shading (T2). Shading weakened the strong positive coupling between the upper and lower canopies observed in the control (*r* = 0.615 to 0.978), shifting specific parameters to an antagonistic relationship under T2 (e.g., lower-canopy *P*_n_ and upper-canopy *L*_s_, *r* = −0.897), which indicates a transition from integrated coordination to functional differentiation. Chlorophyll fluorescence correlations further revealed that while moderate shading (T1) maintained PSII photochemical stability, severe shading (T2) induced divergent energy allocation, with lower-canopy *F*_v_/*F*_m_ negatively associated with upper-canopy qP and NPQ (*r* = −0.361 and −0.355, *p* < 0.05). Furthermore, lower-canopy senescence exerted a 1–2-month time-lagged effect on upper-canopy photosynthesis, with gas exchange parameters demonstrating greater sensitivity than photochemical processes. Overall, the results from three-year-old potted seedlings suggest that shade-induced lower-canopy needle senescence and retention may regulate upper-canopy photosynthetic function, driving an intra-canopy transition from coordination to decoupling and antagonism through distinct time-lagged effects. Further field studies are needed to determine whether these patterns occur in mature *C. lanceolata* stands and how they influence plantation productivity and marcescence dynamics.

## 1. Introduction

Chinese fir (*Cunninghamia lanceolata* (Lamb.) Hook.) is one of the most vital timber species in southern China. Characterized by rapid growth, excellent wood quality, and robust stress resistance, it is widely utilized in construction, furniture manufacturing, and ecological restoration, thereby possessing immense economic value and ecological benefits [1,2]. According to the Ninth National Forest Inventory, the plantation area of Chinese fir has reached 9.90 million hm^2^, with a standing volume of 775 million m^3^, both ranking first among all tree species nationally [3]. Throughout its long evolutionary history, Chinese fir has developed a distinct phenotypic trait of marcescence, defined as the prolonged retention of dead twigs and needles within the canopy. This trait typically becomes apparent around five years of age and can persist for several years [4]. In intensive silviculture of Chinese fir, high-density planting models inevitably generate steep vertical light gradients. Consequently, lower-canopy foliage is subjected to prolonged shading and chronic light limitation, forcing it to senesce gradually and remain attached within the canopy [5,6]. Existing studies indicate that this marcescent biomass is immense, ranging from 6.51 to 9.43 t·ha^−1^ in middle-aged stands and constituting up to 95% of the total aboveground litter in mature forests [4,7]. In fact, marcescence is not unique to Chinese fir; similar foliage retention traits are widely observed in other conifers such as Douglas-fir (*Pseudotsuga menziesii*) and Japanese cedar (*Cryptomeria japonica*) [8,9], as well as broad-leaved genera like oaks (*Quercus* L.) and beeches (*Fagus* L.) [10,11]. Evidently, the senescence and retention of dead foliage represent a key ecological strategy for adapting to canopy light heterogeneity. However, the physiological and ecological functions of these retained tissues, particularly their feedback contributions to whole-canopy productivity, have long been severely underexplored.

Marcescent canopy litter represents a major component of aboveground canopy necromass and acts as a vital driver of carbon and nutrient cycling in forest ecosystems [12,13]. Unlike typical deciduous species, the foliage of marcescent trees remains attached to the main stem or branches for extended periods, creating a uniquely accumulating litter pool within the canopy interior [8,9,14]. Although previous studies on *C. lanceolata* have documented the static biomass distribution of these retained needles [15,16] and their spatio-temporal nutrient dynamics during senescence [17,18], a systematic understanding of how these tissues ecologically and physiologically regulate tree growth and survival remains limited. Mechanistically, self-shading and intraspecific competition within dense forest canopies shape a pronounced vertical light gradient [19,20]. This core environmental driver not only induces plastic differentiation in leaf morphology and physiology [21,22], but also reorganizes the whole-plant functional network through the co-allocation of carbon, water, and nutrients, ultimately triggering massive dead tissue accumulation in the canopy [23,24]. At the organ scale, prolonged low-light stress continuously suppresses the photosynthetic capacity of lower-canopy leaves [25]. This suppression initially manifests as a coordinated decline in both net photosynthetic rate (*P*_n_) and intercellular CO_2_ concentration (*C*_i_), indicating a predominant stomatal limitation that restricts CO_2_ diffusion into the mesophyll [26]. As light stress duration extends, however, the photosynthetic constraint progressively shifts toward non-stomatal limitations. This transition is evidenced by a marked reduction in the maximum photochemical efficiency of PSII (*F*_v_/*F*_m_), weakened non-photochemical quenching (NPQ), and progressive destabilization of the photosystems. Collectively, these photochemical impairments trigger dark-induced leaf senescence (DILS) and ultimately culminate in irreversible photodamage [27,28,29].

Crucially, maximizing plant light-use efficiency under heterogeneous conditions depends on systemic resource optimization rather than localized, autonomous adjustments of individual organs, demonstrating a typical non-autonomous response [30]. To explain how distinct plant organs coordinate within such heterogeneous light environments, two complementary yet opposing theoretical frameworks have emerged, the “correlative inhibition hypothesis” and the “compensatory response hypothesis”. The correlative inhibition hypothesis posits that the senescence of shaded organs exerts a negative, amplifying cascade effect on whole-plant functioning [31,32]. Under chronic low light, restricted resource allocation and light stress act synergistically to suppress carbon, nitrogen, and water assimilation within shaded branches while obstructing their outward transport, thereby accelerating localized functional degradation and magnifying the metabolic drain at the whole-plant scale. Conversely, the compensatory response hypothesis suggests that plants can optimize systemic resource allocation by preferentially directing key limiting nutrients toward high-light (unshaded) foliage [33,34,35]. This target allocation enhances Rubisco carboxylation efficiency and high-light photosynthetic potential, effectively offsetting the carbon deficits of shaded tissues to achieve canopy-scale functional compensation. For instance, an asymmetric, half-canopy shading experiment on 5-year-old *C. lanceolata* seedlings by Dong et al. (2015) revealed a distinct compensatory response, where unshaded branches exhibited enhanced photosynthetic capacity and altered nitrogen allocation as shading intensified, demonstrating robust intra-canopy branch coupling and systemic resource coordination [30]. Evidence from lodgepole pine (*Pinus contorta*) and white spruce (*Picea glauca*) indicates persistent physiological differentiation among crown positions under asymmetric crown shading [36], while studies in Dahurian larch (*Larix gmelinii*) have demonstrated time-dependent translocation, storage, and remobilization of photoassimilates [37].

However, current research remains constrained by short-term or single-branch scales, leaving whole-plant functional restructuring under chronic light heterogeneity ambiguous. Whether the intact canopy shifts from initial integration toward progressive inter-layer decoupling and eventual antagonism, or whether upper foliage sustains enduring metabolic compensatory responses despite lower-canopy suppression remains unresolved. Furthermore, it is unclear whether gas exchange and PSII photochemistry display synchronized or divergent sensitivities to inter-layer signals. To address these gaps, this study subjected three-year-old *C. lanceolata* seedlings to long-term vertical light gradients via lower-canopy shading, comprising half-canopy (moderate, T1) and three-quarter canopy (severe, T2) shading treatments alongside a non-shaded control (CK), to clarify the feedback mechanisms exerted by lower-canopy senescence and marcescent retention on unshaded functional upper-canopy needles. Based on this work, we hypothesized that: (1) chronic lower-canopy shading would progressively impair photosynthetic performance in shaded needles, with shading severity affecting the magnitude of functional deterioration and a shift from early stomatal limitations to late non-stomatal restrictions; (2) prolonged lower-canopy shading would exert retrograde effects on photosynthesis in unshaded upper-canopy needles, driving an intra-canopy functional transition from synergy to progressive decoupling and eventual antagonism, with gas exchange responding more sensitively to vertical light heterogeneity than PSII photochemistry; and (3) the effects of lower-canopy decline may exhibit a distinct ecophysiological time lag, leading to temporal asynchrony and dynamic physiological divergence between canopy layers.

## 2. Results

### 2.1. Effects of Shading on Whole-Plant Growth Dynamics and Biomass Allocation in C. lanceolata Seedlings

#### 2.1.1. Monthly Dynamics of Height and Basal Diameter Increments

Shading differentially affected the longitudinal and radial growth of *C. lanceolata* seedlings (Figure 1). Compared with CK, shading generally enhanced height and basal-diameter growth, particularly under T2, with a stronger relative response in height than in radial growth. For height increment, T2 generally showed higher values and a unimodal pattern, peaking in June at 14.87% and 89.42% above CK and T1, respectively. Height increment declined thereafter, although T1 and T2 remained numerically higher than CK in July and August, with T1 showing relatively small fluctuations. For basal diameter, T1 generally declined after February, whereas T2 remained relatively stable after March and maintained higher values from July to September. Most monthly treatment differences were not significant, except in September, when T2 was significantly higher than CK and T1 (*p* < 0.05).

#### 2.1.2. Root–Shoot Ratio Responses to Shading

Shading significantly reduced the root-to-shoot ratio of *C. lanceolata* seedlings (Figure 2). At the final harvest, the root-to-shoot ratio declined with increasing shading intensity, following the order CK (0.79 ± 0.04) > T1 (0.67 ± 0.05) > T2 (0.47 ± 0.04). The root-to-shoot ratio under T2 was only 59.2% and 70.1% of that in CK and T1, respectively (*p* < 0.05). Collectively, shading prioritized aboveground biomass accumulation over root growth, and this effect intensified as shading severity increased.

### 2.2. Dynamic Effects of Shading on Photosynthesis and Chlorophyll Fluorescence Parameters in Lower-Canopy Needles in C. lanceolata Seedlings

#### 2.2.1. Monthly Dynamics of Photosynthetic Parameters in Lower-Canopy Needles

Shading significantly altered monthly gas exchange dynamics in lower-canopy needles of *C. lanceolata* (Figure 3). During the experiment, *P*_n_ was generally suppressed under T1 and T2, particularly from March onward, whereas other gas-exchange parameters showed marked temporal variation. From January to February, *P*_n_, *G*_s_, and *C*_i_ decreased significantly, while *L*_s_ increased, indicating enhanced stomatal restriction. In March, however, *P*_n_ remained lower under shading, whereas *G*_s_ and *C*_i_ partially recovered and *L*s declined. From April to May, *P*_n_ in T1 and T2 peaked in April and then declined rapidly thereafter, while *C*_i_ reached maxima in May (T1: 382.33; T2: 391.00 μmol·mol^−1^) and *L*_s_ remained relatively low, suggesting increasing non-stomatal constraints. In June, *P*_n_, *G*_s_, and *E* were markedly suppressed under both shading treatments, whereas T1 and T2 showed contrasting responses in *C*_i_, *L*_s_, and WUE. T1 exhibited lower *C*_i_ and higher *L*_s_, whereas T2 showed higher *C*_i_, lower *L*_s_, and the lowest WUE. Overall, prolonged shading impaired photosynthetic performance, with stomatal and non-stomatal limitations varying among months.

#### 2.2.2. Monthly Dynamics of Chlorophyll Fluorescence Parameters in Lower-Canopy Needles

Shading significantly affected chlorophyll fluorescence parameters of lower-canopy needles in Chinese fir and revealed a staged progression of PSII damage (Figure 4). The maximum photochemical efficiency (*F*_v_/*F*_m_) peaked in February (0.80–0.82) across all treatments, after which CK remained high while T1 and T2 declined significantly from May, reaching 0.63 and 0.51 in June, respectively (*p* < 0.05). This indicated a shift from reversible stress to irreversible PSII damage and identified May–June as the critical period of shading-induced senescence. The effective photochemical efficiency (Y(II)) increased to a June peak (0.28) in CK but remained consistently lower and significantly suppressed in T1 and T2. Non-photochemical quenching (NPQ) peaked in March and then declined, with T2 dropping to 41% of CK by June, suggesting impaired thermal dissipation, while photochemical quenching (qP) was approximately 0.52 in both CK and T1, but was significantly lower in T2. Energy-partitioning parameters further supported this pattern. Both Y(NO) and Y(NPQ) were significantly affected by shading treatment and month. By June, T2 showed a marked increase in Y(NO) accompanied by a significant decline in Y(NPQ) (Figure S1).

### 2.3. Dynamic Effects of Shading on Photosynthesis and Chlorophyll Fluorescence Parameters in Upper-Canopy Needles in C. lanceolata Seedlings

#### 2.3.1. Monthly Dynamics of Photosynthetic Parameters in Upper-Canopy Needles

To characterize longer-term changes in upper-canopy photosynthetic performance, monthly gas-exchange measurements were used to assess temporal variation across the experimental period (Figure 5). Net photosynthetic rate (*P*_n_) increased to a June maximum in CK (5.13 μmol CO_2_·m^−2^·s^−1^), whereas T1 and T2 peaked earlier in May and generally declined thereafter, with a transient increase in August; by September, T2 had declined to 38.0% of CK (*p* < 0.05) (Figure 5a). Stomatal conductance (*G*_s_) and transpiration rate (*E*) showed similar trends, both peaking in August, with T1 highest (*G*_s_: 200.67 mmol H_2_O·m^−2^·s^−1^; *E*: 4.28 mmol·m^−2^·s^−1^), followed by CK and then T2 (Figure 5b,e). Intercellular CO_2_ concentration (*C*_i_) and stomatal limitation (*L*_s_) displayed significant inverse patterns (Figure 5c,f): in July, T1 showed low *C*_i_ and high *L*_s_, whereas T2 exhibited high *C*_i_ and low *L*_s_, suggesting a shift in photosynthetic limitation from stomatal to non-stomatal factors. Water use efficiency (WUE) also varied markedly among months and treatments and reached its lowest value under T1 in August (0.92) (Figure 5d). Overall, lower-canopy shading markedly altered the temporal gas-exchange dynamics of unshaded upper-canopy needles.

#### 2.3.2. Diurnal Dynamics of Photosynthetic Parameters in Upper-Canopy Needles

As a complementary assessment of short-term physiological variation, the July diurnal measurements revealed treatment-dependent differences in the photosynthetic responses of unshaded upper-canopy needles (Figure 6). *P*_n_ generally declined throughout the day, although T1 showed a transient increase at 10:00 and was significantly higher than CK and T2 at this time point (*p* < 0.05). *G*_s_ declined progressively in CK, showed peaks at 10:00 and 16:00 in T1, and remained relatively low in T2 after an early-morning maximum. *C*_i_ showed corresponding fluctuations, particularly in T1 and T2. WUE peaked at 16:00 in CK and 14:00 in T1, whereas T2 remained comparatively low. *E* generally declined in CK and T2 but showed two peaks in T1. *L*_s_ peaked at 14:00 in CK and T1 and at 12:00 in T2.

#### 2.3.3. Monthly Dynamics of Chlorophyll Fluorescence Parameters in Upper-Canopy Needles

Chlorophyll fluorescence parameters of unshaded upper-canopy needles were significantly influenced by lower-canopy shading (Figure 7). *F*_v_/*F*_m_ showed a rise-decline pattern across treatments, peaking in February (0.81–0.83) and then decreasing, with T1 and T2 declining significantly from July and reaching minima in August (T1: 0.56; T2: 0.53), both lower than CK (0.63; *p* < 0.05) (Figure 7a). Y(II) remained relatively stable at 0.23–0.29 during March–May before declining; T2 peaked at 0.29 in May, exceeded CK and T1 from May to July, and then dropped markedly during July–August (Figure 7b). NPQ differed significantly among treatments, with T1 generally higher than T2; T2 peaked in May (3.22) and declined to 1.94 by August (Figure 7c). qP fluctuated between 0.26 and 0.52, with T2 exceeding CK in May and July but declining in August in parallel with *F*_v_/*F*_m_ (Figure 7d). Additional fluorescence parameters (Figure S2) further supported these patterns. Y(NPQ) and Y(NO) exhibited marked temporal fluctuations, particularly under T2, indicating dynamic adjustments in regulated and non-regulated energy dissipation across the experimental period.

### 2.4. Functional Coupling and Time-Lagged Regulation Between Upper and Lower Canopy in C. lanceolata Seedlings

#### 2.4.1. Progressive Decoupling of Gas-Exchange Traits with Intensified Shading

Correlation analysis (Table 1) showed that shading progressively weakened canopy coupling and induced structural reorganization of their interrelationships. Strong positive correlations in CK (*r* = 0.615**–0.978**) indicated coordinated gas exchange between layers, whereas T1 showed reduced coupling and a shift of *P*_n_ from positive to negative correlation, suggesting a transition from coordination to competition. In T2, most relationships became non-significant, indicating functional decoupling between canopy layers. Notably, a strong negative correlation between lower-canopy *P*_n_ and upper-canopy *L*_s_ persisted (*r* = −0.897**, *p* < 0.01), reflecting an antagonistic relationship. Overall, increasing shading intensity drives an intra-canopy functional transition from synergy to competition and ultimately to decoupling.

#### 2.4.2. Contrasting Stability Between Photochemical Integration and Spatial Differentiation in Energy Dissipation

Fluorescence correlations (Table 2) indicated photochemical coupling remained relatively stable compared with gas-exchange traits. Across all treatments, *F*_v_/*F*_m_ remained strongly positively correlated between canopy layers, and Y(II) coupling strengthened with increasing shading intensity, reflecting conserved photochemical integration within the canopy. However, functional divergence emerged in T2, with negative correlations between lower-canopy *F*_v_/*F*_m_ and upper-canopy qP and NPQ, indicating enhanced thermal dissipation and antagonistic energy allocation. Overall, photochemistry remained coordinated across canopy layers, whereas energy dissipation became spatially differentiated under strong shading stress.

#### 2.4.3. Time-Lagged Effects of Lower-Canopy Senescence on Upper-Canopy Needles

Time-lag correlation analysis (Table 3) revealed significant temporal associations between lower-canopy needle senescence (May–June) and subsequent upper-canopy physiological variation (June–September). In T1, positive correlations between lower- and upper-canopy *P*_n_ and *G*_s_ were consistent with sustained stomatal–carbon coordination, while the negative correlation between lower-canopy *F*_v_/*F*_m_ and upper-canopy Y(II) indicated an inverse time-lagged association in PSII photochemical responses. In T2, stronger inverse fluorescence relationships were observed, with significant negative correlations between lower-canopy NPQ and *F*_v_/*F*_m_ and upper-canopy qP, suggesting greater temporal divergence in photochemical regulation between canopy layers. Overall, these results indicate time-lagged associations between lower-canopy decline and subsequent upper-canopy physiological variation, with the correlation patterns differing between shading treatments.

## 3. Discussion

### 3.1. Shading-Accelerated Senescence and Functional Collapse in Lower-Canopy Needles from Stomatal to PSII Impairment

This study supports our first hypothesis that prolonged shading accelerates senescence of lower-canopy needles in Chinese fir, with a clear intensity-dependent response. Under T1 and T2 treatments, lower-canopy needles exhibited visible yellowing in May, extensive chlorosis in June, and persistent retention from July to September, whereas CK needles maintained functional status for longer periods. This accelerated senescence is consistent with previous studies showing that chronic low-light conditions induce premature leaf senescence through carbon assimilation constraints and disrupted carbon–nutrient balance [38,39,40]. However, in marcescent conifers such as Chinese fir, senescence may not represent an immediate resource reallocation. Because dead needles remain attached to branches, their persistence can further reduce local light availability, generating a reinforcing “shading–senescence–re-shading” feedback that accelerates functional deterioration of lower-canopy tissues. This mechanism fundamentally differs from deciduous species, where rapid leaf abscission effectively terminates the carbon cost of senescent tissues.

The coordinated responses of *P*_n_, *G*_s_, *C*_i_, *E*, and *L*_s_ indicate a progressive transition from stomatal regulation to biochemical impairment during shade-induced senescence. During the early stages of shading, reductions in *P*_n_ accompanied by declines in *G*_s_ and *C*_i_, and increased *L*_s_ (Figure 3a–c,f), suggested that restricted CO_2_ diffusion caused by stomatal closure represented the primary limitation. During the later stages of shading, *P*_n_ declined markedly in both T1 and T2, while *C*_i_ increased in May and remained relatively high in T2 in June (Figure 3a,c), suggesting an increasing contribution of non-stomatal limitations, particularly under severe shading [41,42,43]. This biphasic transition has been reported in shade-stressed woody seedlings [44]. However, the earlier onset and greater decline under T2 in our study indicate limited acclimative plasticity in Chinese fir when light deprivation is prolonged. Moreover, the sustained reduction in WUE under T1 and T2 (Figure 3d) indicates that shading simultaneously impaired carbon gain and stomatal regulation, resulting in progressive resource-use inefficiency. This finding corroborates and extends the localized shading results of Dong et al. [30] on lateral branches of 5-year-old Chinese fir seedlings.

Chlorophyll fluorescence parameters further characterized the response of PSII to prolonged shading in lower-canopy needles. Healthy leaves typically maintain *F*_v_/*F*_m_ values of 0.75–0.85, with lower values indicating reduced PSII efficiency or increased photochemical stress [45]. The decline in *F*_v_/*F*_m_ under shading, particularly under T2 during the late experimental period (Figure 4a), suggested reduced PSII photochemical stability under persistent low irradiance and seasonal environmental stress, consistent with responses reported in evergreen species under winter low-light conditions [46]. Although the temporary recovery of *F*_v_/*F*_m_ in February indicated partial reversibility, prolonged shading was associated with altered thylakoid energy regulation and photoprotective capacity [47,48]. Notably, qP remained relatively stable under T1 despite the late-stage decline in *F*_v_/*F*_m_, suggesting that widespread reaction-center closure was not the dominant response; rather, PSII efficiency declined while the remaining reaction centers retained a relatively high degree of openness. The subsequent decline in Y(II) (Figure 4b) further indicated reduced PSII photochemical efficiency during the later experimental period. Meanwhile, the initial increase in NPQ (Figure 4c) reflected enhanced thermal dissipation as a protective adjustment [49], whereas its subsequent decline under severe shading indicated reduced photoprotective capacity under prolonged shading [50]. Consistently, increased Y(NO) together with decreased Y(NPQ) under T2 by June indicated a shift from regulated photoprotective dissipation toward greater non-regulated energy loss (Figure S1). This low-light response contrasts with high-light photoinhibition reported in the evergreen conifer *Abies sachalinensis*, which involves increased PSII excitation pressure and altered energy dissipation [51]. In our study, prolonged shading was instead associated with reduced PSII photochemical stability and photoprotective capacity, while the underlying mechanisms remain unclear. This contrast may provide insight into shade-acclimation responses in evergreen conifers.

### 3.2. Senescence of Lower-Canopy Needles Drives a Shift in Canopy Photosynthetic–Fluorescence Coordination from Integration to Functional Differentiation

This study supports our second hypothesis that shade-induced functional decline in lower-canopy needles progressively suppresses upper-canopy photosynthesis through cross-layer regulation, resulting in a progressive transition from synergistic coupling to functional decoupling and, ultimately, antagonistic interactions with increasing shading intensity. Critically, we found that treatment effects on gas exchange emerged earlier than pronounced PSII photochemical changes, suggesting earlier stomatal adjustment and comparatively delayed photochemical responses.

This differential sensitivity was evident at the monthly scale. The decline in upper-canopy *P*_n_ was accompanied by treatment- and month-dependent changes in *G*_s_, *C*_i_, and *L*_s_ (Figure 5), suggesting that stomatal regulation and restricted CO_2_ diffusion contributed to the early decline in photosynthesis [52,53]. Importantly, *P*_n_ declined under T1 and T2 before visible lower-canopy senescence, indicating an early whole-plant physiological adjustment rather than a direct consequence of senescence. Prolonged lower-canopy shading may alter source–sink carbon balance and assimilate partitioning [54], while canopy-level photosynthetic acclimation associated with light perception and physiological signaling may occur before visible senescence develops [55]. These responses are consistent with cross-layer physiological coordination within the crown. Notably, upper-canopy needles exhibited a transient recovery of *P*_n_ in August, coinciding with increased *G*_s_ and *E* (Figure 5b,e) rather than improved photochemical efficiency. This pattern suggests a short-term compensatory adjustment through enhanced gas exchange [56,57]. However, the concurrent decline in WUE indicates a trade-off between carbon gain and water conservation. Previous studies have shown that stomatal and photosynthetic responses may become decoupled under changing light, such that increased stomatal opening does not necessarily produce a proportional increase in carbon assimilation [58,59]. Accordingly, the relatively high *G*_s_ and *C*_i_ in T1 at 10:00 may reflect transient stomatal opening rather than reduced carbon fixation. This response may involve guard-cell light signaling [60], while the asynchronous regulation of stomatal conductance and photosynthesis may also contribute to this pattern. In contrast, PSII photochemistry showed a comparatively delayed response. Increased Y(II) and qP from March to July (Figure 7) suggested relatively stable PSII function, whereas declines in *F*_v_/*F*_m_, Y(II), and NPQ in August indicated reduced photochemical stability and photoprotective capacity. This temporal sequence suggests a multi-stage canopy adjustment in which earlier stomatal regulation may represent an initial physiological response associated with lower-canopy shading, whereas later photochemical changes were associated with subsequent functional decline [61,62,63].

Correlation analysis further revealed a progressive reorganization of carbon–water–energy coupling among canopy layers. Under CK conditions, strong positive correlations among *P*_n_, *E*, and WUE indicated coordinated regulation across canopy positions [64]. Under moderate shading (T1), these relationships weakened, particularly between *P*_n_ and *E* (Table 1), suggesting reduced synchronization of carbon gain and water regulation between canopy layers [65]. Under severe shading (T2), the loss of coordination among gas-exchange parameters indicated that canopy carbon acquisition became increasingly constrained by altered physiological regulation and structural heterogeneity [66]. A similar transition occurred in PSII regulation. Chlorophyll fluorescence parameters shifted from coordinated variation under CK to weakened or negative correlations under T2 between canopy layers (Table 2). This indicates that energy allocation strategies gradually shifted from coordinated regulation toward functional differentiation between canopy layers. The lower canopy tended to enhance thermal dissipation through NPQ, whereas the upper canopy maintained higher photochemical utilization capacity through qP regulation, suggesting divergent energy-management strategies under persistent stress [45,67]. Thus, canopy stability under persistent shading does not rely solely on unidirectional compensation but involves dynamic reorganization of carbon–water–energy relationships, in which the lower canopy first experiences stomatal limitation, and this signal propagates upward to alter upper-canopy regulation through cross-layer interactions, ultimately disrupting canopy-scale functional coordination and driving the system toward vertical functional differentiation.

### 3.3. Time-Lagged Inhibition and Ecological Implications of Persistent Marcescent Retention

This study supports our third hypothesis that persistent retention of senescent lower-canopy needles contributes to canopy functional reorganization through delayed vertical interactions between canopy layers. Time-lag analysis (Table 3) revealed that functional decline of lower-canopy needles during May–June was significantly associated with subsequent physiological changes in upper-canopy needles from June to September. Moreover, temporal coupling was stronger for gas exchange parameters (*P*_n_ and *G*_s_) than for fluorescence parameters, suggesting that canopy regulation follows a hierarchical response sequence, in which carbon acquisition and stomatal processes respond earlier, whereas photochemical adjustments occur after cumulative light-deprivation stress exceeds a physiological threshold. Mechanistically, the observed 1–2-month time lag likely involves long-distance signaling cascades, including systemic transport of phytohormones (e.g., abscisic acid, cytokinins) and sugar-mediated source–sink feedback [68,69]. Senescing lower needles may alter carbohydrate export and hormone profiles in the transpiration stream, which, upon reaching upper needles, progressively modulate stomatal conductance and, subsequently, photochemical efficiency. Although direct evidence for such vertical signaling in woody canopies remains limited, our correlational data provide strong inferential support for this hypothesis, extending the concept of temporal hysteresis from individual-leaf dynamics to whole-canopy inter-layer regulation [70].

With increasing shading intensity, inter-layer relationships shifted progressively from coordination toward functional divergence, reinforced by delayed physiological responses. Under moderate shading (T1), significant positive correlations between lower- and upper-canopy *P*_n_ and *G*_s_ indicated the maintenance of stomatal–carbon coordination during early shading. This pattern is consistent with previous studies showing that stomatal regulation can constrain photosynthesis through restricted CO_2_ diffusion under stress [43]. However, the emergence of negative relationships between lower-canopy *F*_v_/*F*_m_ and upper-canopy Y(II) suggested that photochemical regulation became increasingly decoupled as stress accumulated [45]. Under severe shading (T2), this divergence became more pronounced. The negative associations between lower-canopy NPQ, *F*_v_/*F*_m_ and upper-canopy qP indicated that contrasting energy-use patterns occurred between canopy layers. Higher NPQ generally reflects enhanced regulated thermal dissipation and photoprotection [71], whereas variation in qP reflects PSII reaction-center openness and photochemical energy utilization [72]. Thus, this pattern is consistent with greater reliance on thermal dissipation in the lower canopy and relatively greater photochemical utilization in the upper canopy. Critically, these results suggest that canopy regulation reflects accumulated physiological effects rather than instantaneous responses alone, with a shift from coordinated regulation toward functional specialization and increasing biochemical and photochemical constraints. The observed pattern of lower-canopy decline with temporary upper-canopy maintenance further suggests that evergreen conifers may preserve whole-canopy carbon gain by prioritizing functional maintenance of higher-efficiency needles under stress [70]. However, when lower-canopy degradation exceeds the compensatory capacity of the canopy, accumulated lag effects may eventually disrupt canopy-scale coordination and accelerate functional decoupling.

The persistent marcescent retention observed in Chinese fir embodies a double-edged ecological strategy. On one hand, moderate retention may help maintain canopy structure and buffer environmental fluctuations. On the other hand, excessive accumulation under prolonged shading creates a self-reinforcing feedback that accelerates lower-canopy functional decline and progressively constrains whole-plant carbon assimilation. From an applied perspective, our findings suggest that sustainable management of Chinese fir plantations should balance canopy retention with functional renewal. Specifically, silvicultural practices such as targeted pruning of senescent or heavily shaded lower branches and timely thinning interventions to improve vertical light penetration could effectively interrupt the “shading–senescence–re-shading” feedback. Additionally, early-season monitoring of *F*_v_/*F*_m_ and *P*_n_ relationships between canopy layers may provide an early warning of functional decoupling and support timely intervention. However, these implications are based on three-year-old potted seedlings and should not be directly extrapolated to mature field-grown *C. lanceolata* plantations. Stand-level validation is therefore required before these strategies can be broadly applied.

## 4. Materials and Methods

### 4.1. Plant Materials and Experimental Site

Three-year-old seedlings of *C*. *lanceolata* were selected as experimental materials and cultivated in the intensive forest breeding greenhouse of Fujian Agriculture and Forestry University. The experiment was carried out in Fuzhou, China (26°05′ N, 119°18′ E). During the experimental period, ambient air temperature ranged from 18 to 32 °C, and relative humidity ranged from 65% to 80%. Regular quantitative irrigation was applied according to seedling growth performance to exclude drought-induced interference with experimental measurements.

### 4.2. Experimental Design

The experiment was conducted from January to September 2024. Nine uniform, three-year-old *Cunninghamia lanceolata* seedlings were selected and cultivated individually in pots containing a mixed substrate of yellow soil and peat (1:1, *v*/*v*) (Table 4). The seedlings were randomly assigned to three treatments with three replicates each: the unshaded control (CK), moderate shading treatment (T1), and severe shading treatment (T2). Both T1 and T2 used two layers of 30-pin shading netting, and measurements with a portable spectrometer (HR-350, Taiwan HiPoint Corporation, Taiwan, China) showed that the mean illuminance was 2140 lx under unshaded conditions and 65.0 lx within the shaded zones, corresponding to 96.96% attenuation and 3.04% transmittance. Specifically, the shading net covered the lower 1/2 of the aboveground plant height in T1 and the lower 3/4 height in T2, whereas the upper branches in both treatments remained fully exposed to natural ambient light (Figure 8). During the experimental period, healthy branches from the second branch whorl (shaded zone) and the fourth branch whorl (unshaded zone) counted from the plant base were selected and tagged for the dynamic monitoring of seedling growth indicators and needle photosynthetic characteristics.

### 4.3. Determination of Growth and Biomass Traits

Seedling height and ground diameter were measured monthly, with monthly increments derived from paired measurements. All seedlings were destructively harvested at the end of the experiment. Control seedlings were separated into eight tissue fractions, whereas shaded seedlings were cut at the shading boundary to isolate upper healthy and lower senescent tissues; roots of all plants were categorized as fine, medium or coarse. All tissues were sequentially rinsed with tap water, detergent, 1% HCl and distilled water, oven-dried (105 °C for 30 min deactivation, 80 °C to constant mass) to weigh organ dry biomass. The root-to-shoot ratio was determined only at the final harvest to avoid repeated root disturbance, and thus reflects end-point differences in biomass allocation. The root-to-shoot ratio (*R*/*S*), a commonly used indicator of whole-plant biomass allocation [73], was calculated as follows:

(1) $$\frac{R}{S}\;=\;\frac{B_{\mathrm{below}}}{B_{\mathrm{above}}}$$

where *R*/*S* is the root-to-shoot ratio, *B*_above_ represents aboveground biomass, and *B*_below_ represents belowground biomass.

### 4.4. Measurement of Photosynthetic Parameters

Leaf gas exchange parameters were measured using a portable photosynthesis system (PP Systems, Amesbury, MA, USA). During measurements, the CO_2_ concentration in the leaf chamber (*C*_a_) was maintained at 400 μmol·mol^−1^ using the built-in CO_2_ mixing system, leaf temperature was controlled at 25 °C, and relative humidity was kept at 60–70%. Fully expanded and healthy needles were selected from designated whorl branches for each measurement, avoiding both apical buds and basal needles. For gas-exchange measurements, three seedlings from each treatment were selected as independent biological replicates (*n* = 3). At each sampling time, one needle sample was measured from each designated canopy position of each seedling, resulting in three needle samples per treatment and canopy position.

Monthly dynamics of photosynthetic parameters were assessed once per month during the last ten days of each month on clear days between 08:00 and 12:00. Measurements were conducted on healthy needles from the 2nd (lower canopy) and 4th (upper canopy) whorl branches of *Cunninghamia lanceolata* under each treatment. Lower-canopy needles were measured monthly from January to June (3 treatments × 3 seedlings × 6 months), whereas upper-canopy needles were measured from January to September (3 treatments × 3 seedlings × 9 months). The measured parameters included net photosynthetic rate (*P*_n_, μmol CO_2_·m^−2^·s^−1^), intercellular CO_2_ concentration (*C*_i_, μmol·mol^−1^), transpiration rate (*E*, mmol·m^−2^·s^−1^), and stomatal conductance (*G*_s_, mmol H_2_O·m^−2^·s^−1^). Stomatal limitation (*L*_s_) [74] and water use efficiency (WUE) were calculated using the following equations:

(2) $$L_{s}\;=\;1\;-\;\frac{C_{i}}{C_{a}}$$

(3) $$\mathrm{WUE}\left(\mathrm{mmol}\;{\mathrm{CO}}_{2}\;{\mathrm{mol}}^{-1}\;H_{2}O\right)\;=\;\frac{P_{n}}{E}$$

where *C*_a_ denotes the ambient CO_2_ concentration within the leaf chamber.

Notably, lower needles lost detectable photosynthetic activity after July, marking irreversible senescence. Thus, January–June was defined as their functional survival window, with measurements spanning this period for lower foliage and January–September for upper needles.

To characterize short-term within-day variation in upper-canopy photosynthetic physiology, diurnal gas exchange was measured at 2-h intervals from 08:00 to 18:00 on 22 July 2024. Photosynthetic indices of needles from the upper fourth whorl were measured across all treatments using the methods described above.

### 4.5. Measurement of Chlorophyll Fluorescence Parameters

The chlorophyll fluorescence of needles from the 2nd (lower) and 4th (upper) canopy whorls was measured monthly (late month, 08:00–12:00, sunny days) with a Yaxin-1165 fluorometer (Beijing Yaxin, China). Three seedlings per treatment were used as independent biological replicates (*n* = 3), with one needle sample measured per canopy position and seedling at each sampling date. Lower-canopy needles were measured from January to June (3 treatments × 3 seedlings × 6 months) and upper-canopy needles from January to September (3 treatments × 3 seedlings × 9 months).

For dark-adapted assays, needles were dark-adapted for 20 min. Minimal fluorescence (*F*_o_) was captured under 0.03 μmol·m^−2^·s^−1^ modulated light; then a 0.8 s saturating pulse (8000 μmol·m^−2^·s^−1^) yielded maximum fluorescence (*F*_m_). PSII maximum photochemical efficiency was calculated: *F*_v_/*F*_m_ = (*F*_m_ − *F*_o_)/*F*_m_.

For light-adapted measurements, needles were exposed to 800 μmol·m^−2^·s^−1^ actinic light for 5 min to measure steady-state fluorescence (*F*_s_); light-adapted maximum fluorescence (*F*_m_′) and light-adapted minimal fluorescence (*F*_o_′) were sequentially determined. Multiple fluorescence parameters were calculated: the effective quantum yield of PSII (*F*_v_′/*F*_m_′), relative electron transport rate (rETR), photochemical quenching coefficient (qP), non-photochemical quenching coefficient (qN), non-photochemical quenching (NPQ), actual photochemical efficiency of PSII (Y(II)), quantum yield of regulated non-photochemical energy loss (Y(NPQ)), and quantum yield of unregulated non-photochemical energy loss (Y(NO)).

### 4.6. Statistical Analyses

One-way ANOVA and an independent-samples *t*-test detected inter-treatment differences at *p* < 0.05. Pearson correlation quantified cross-canopy associations of photosynthetic and fluorescence traits, with all data presented as mean ± standard error (SE). January–June datasets were used to analyze vertical canopy coupling, as lower needles lost activity after July. Time-lag correlations between lower needle senescence indicators (May–June) and upper foliage physiological variables (June–September) were further analyzed to reveal delayed upper-canopy responses to lower-layer senescence.

## 5. Conclusions

This study demonstrates that persistent retention of senescent lower-canopy biomass can influence the functional organization of *C. lanceolata* seedlings through delayed cross-layer physiological responses. A key finding of this study is that localized lower-canopy shading was associated with subsequent physiological changes in unshaded upper-canopy needles, revealing temporal asynchrony and functional coordination between canopy layers. With increasing shading intensity, cross-layer interactions shift from compensatory coordination toward functional constraints, accompanied by a transition in photosynthetic limitation from stomatal regulation to non-stomatal restrictions, suggesting an increasing role of photosystem impairment and energy dissipation in regulating canopy carbon gain during advanced stages of canopy decline. These findings reveal a structural differentiation strategy in which preferential senescence and retention of lower-canopy tissues may contribute to short-term canopy persistence and stability, but potentially constrain long-term photosynthetic capacity and carbon acquisition under prolonged shading conditions. Because these results were obtained from seedling-scale experiments, stand-level field validation is required. Future studies should integrate multi-season measurements of vertical light distribution, canopy structure and physiology, source–sink resource redistribution, and time-lagged physiological responses across contrasting stand densities and management regimes before these cross-layer physiological responses are incorporated into forest growth or carbon-cycle models.

## Figures and Tables

**Figure 1:**
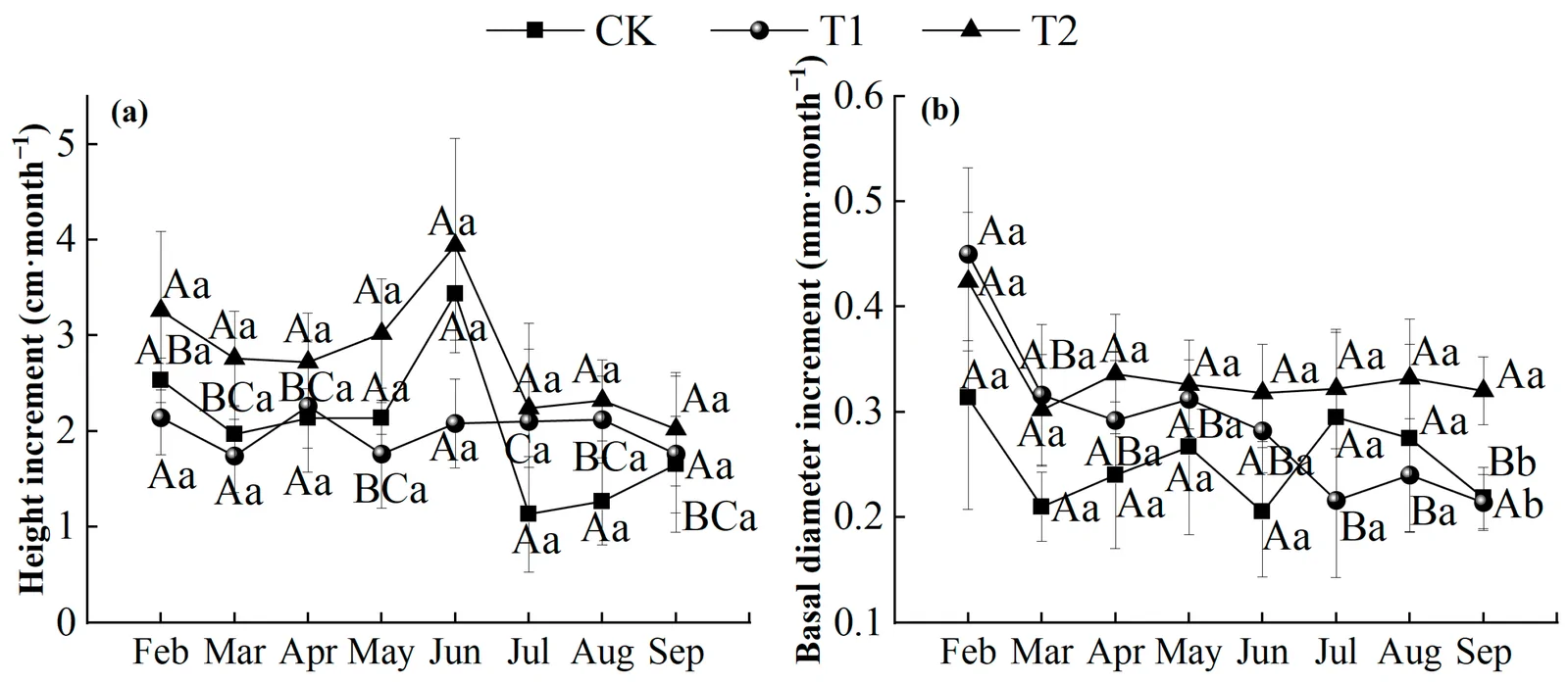
Effects of shading treatments on height and basal-diameter increments of *Cunninghamia lanceolata* seedlings. (**a**) Height increment; (**b**) Basal diameter increment. CK: control; T1: moderate shading; T2: severe shading. Note: Different uppercase letters indicate significant differences between months under the same treatment (*p* < 0.05). Different lowercase letters indicate significant differences between treatments in the same month (*p* < 0.05). Error bars represent standard error (*n* = 3).

**Figure 2:**
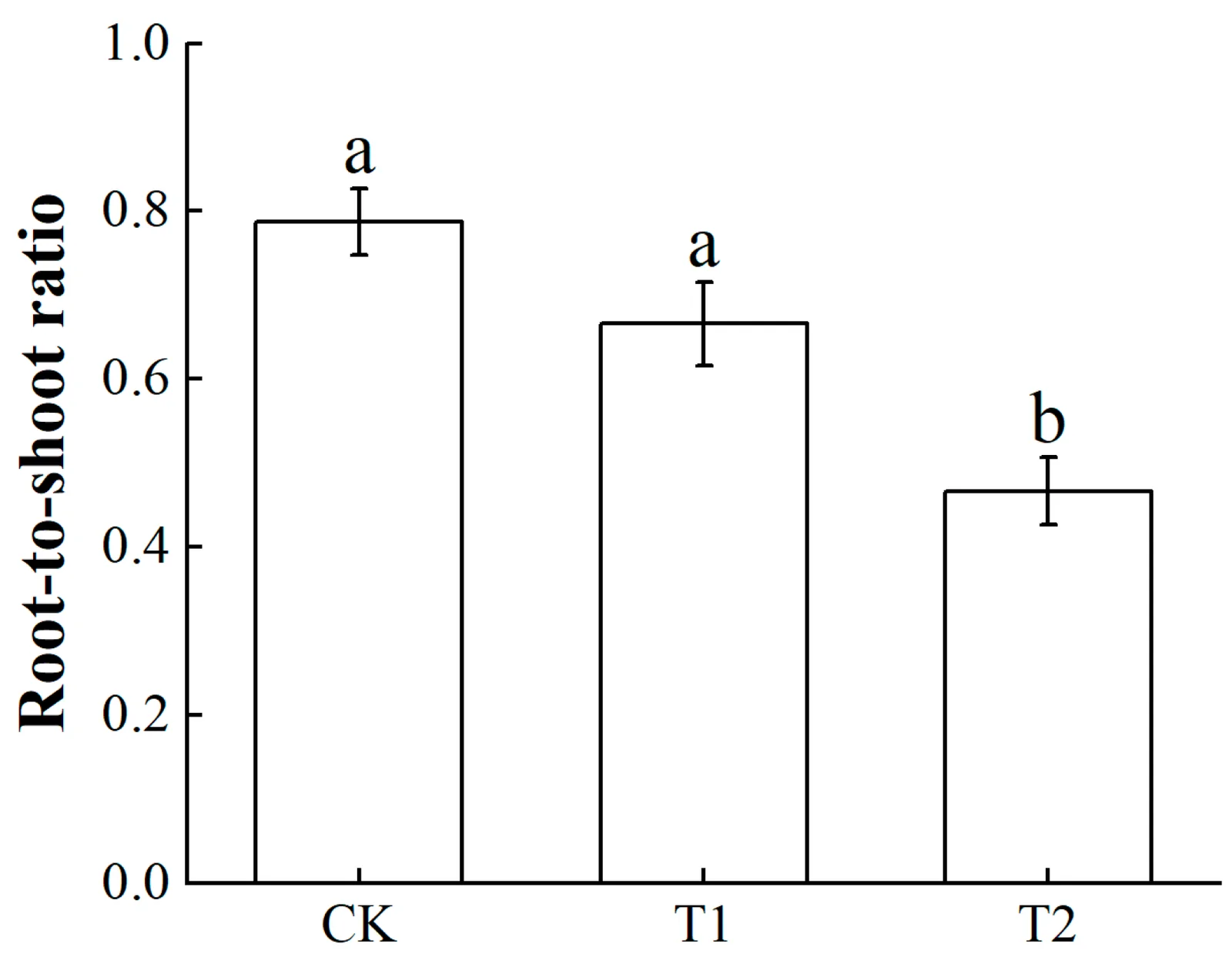
Differences in root–shoot ratios of Chinese fir between different treatments. CK: control; T1: moderate shading; T2: severe shading. Note: Different lowercase letters indicate significant differences among treatments (*p* < 0.05). Error bars represent standard error (*n* = 3).

**Figure 3:**
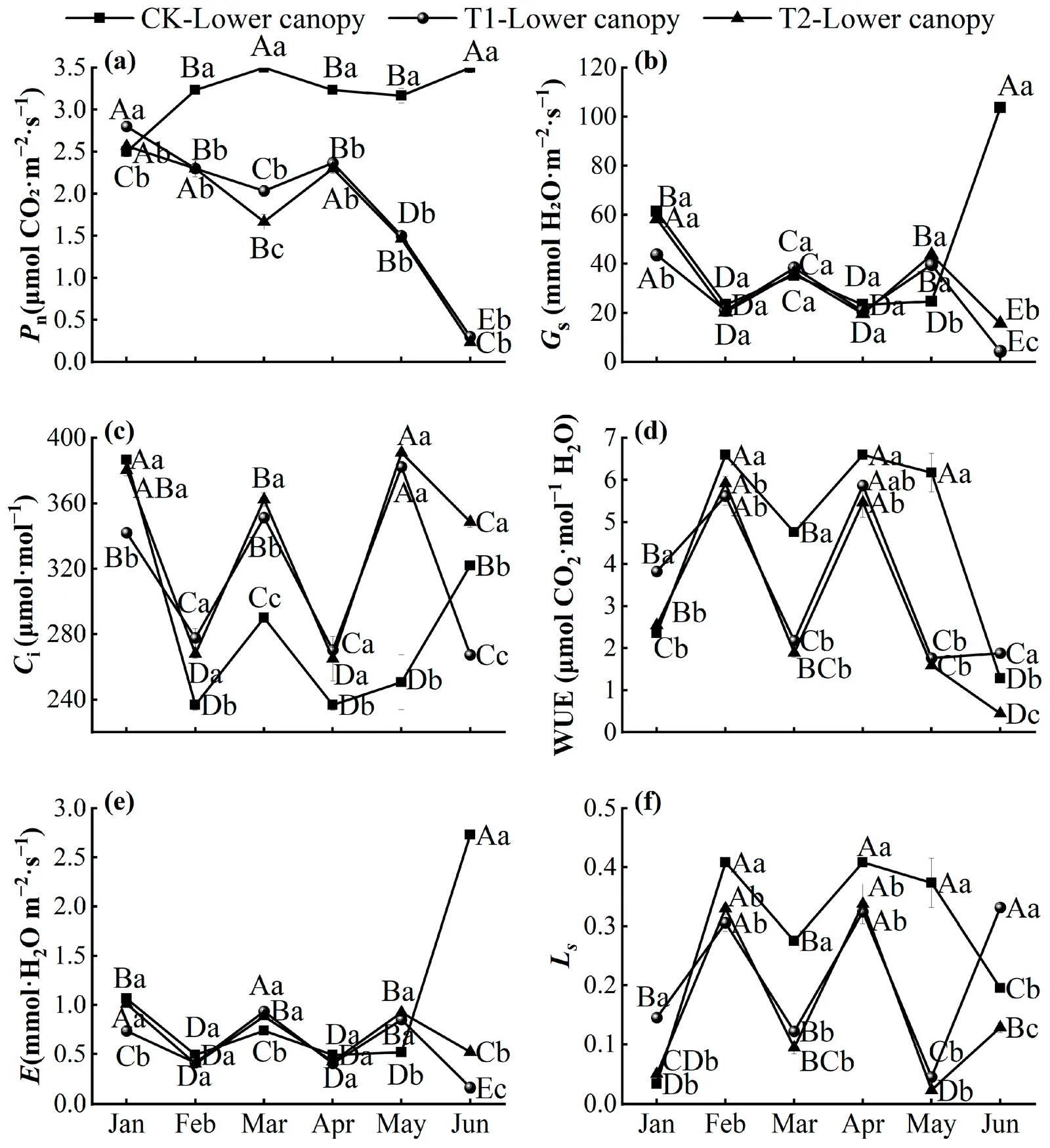
Monthly dynamics of photosynthetic parameters in shaded lower-canopy needles of Chinese fir. (**a**) Net photosynthetic rate (*P*_n_); (**b**) Stomatal conductance (*G*_s_); (**c**) Intercellular CO_2_ concentration (*C*_i_); (**d**) Water use efficiency (WUE); (**e**) Transpiration rate (*E*); (**f**) Stomatal limitation (*L*_s_). CK: control; T1: moderate shading; T2: severe shading. Note: Different uppercase letters indicate significant differences among months within the same treatment (*p* < 0.05); different lowercase letters indicate significant differences among treatments in the same month (*p* < 0.05). Error bars represent standard error.

**Figure 4:**
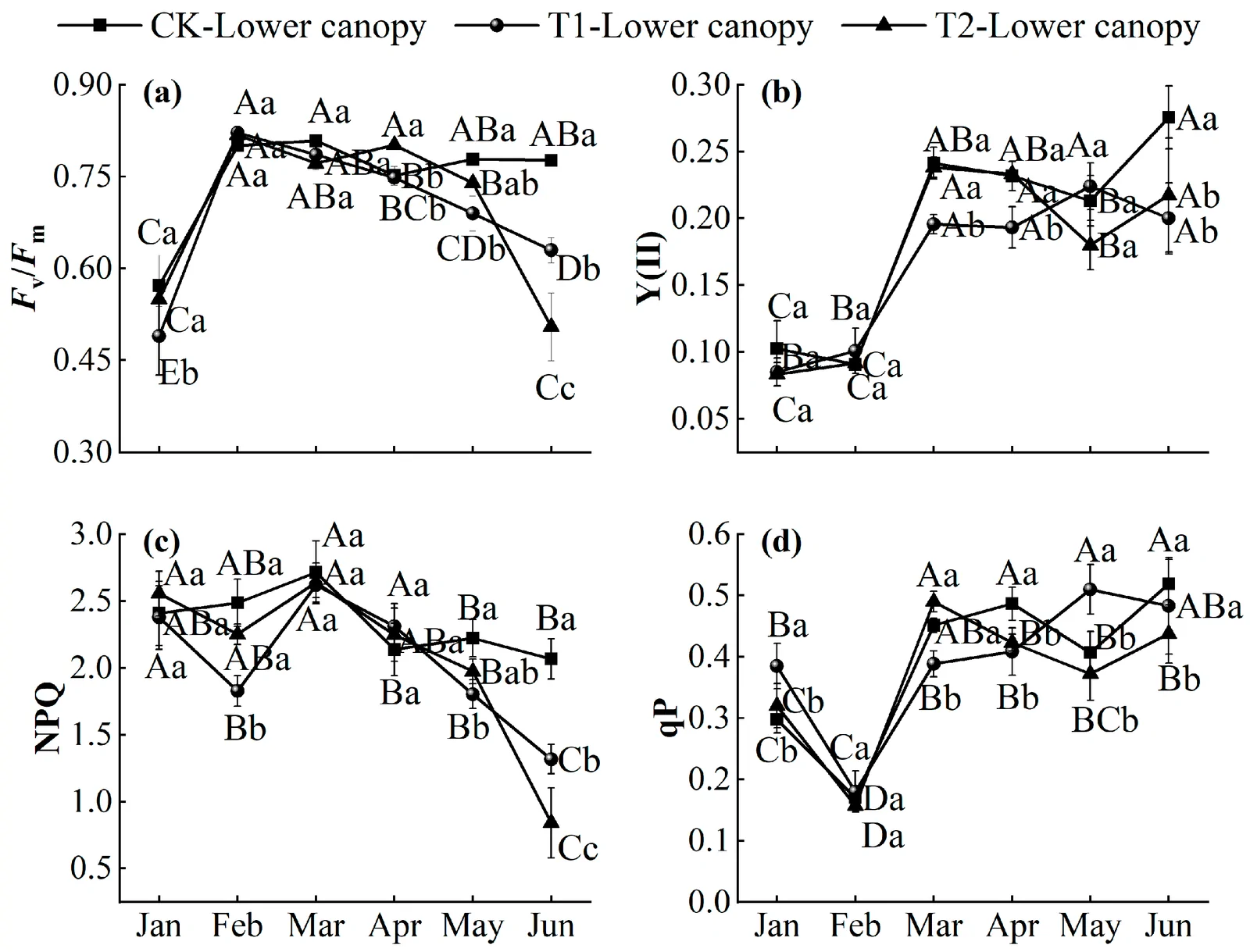
Monthly dynamics of key chlorophyll fluorescence parameters in shaded lower-canopy needles of Chinese fir. (**a**) Maximum photochemical efficiency of photosystem II (*F*_v_/*F*_m_); (**b**) Actual photochemical efficiency of PSII (Y(II)); (**c**) Non-photochemical quenching (NPQ); (**d**) Photochemical quenching (qP). CK: control; T1: moderate shading; T2: severe shading. Note: Different uppercase letters indicate significant differences among months within the same treatment (*p* < 0.05); different lowercase letters indicate significant differences among treatments in the same month (*p* < 0.05). Error bars represent standard error. The complete dataset is provided in Figure S1.

**Figure 5:**
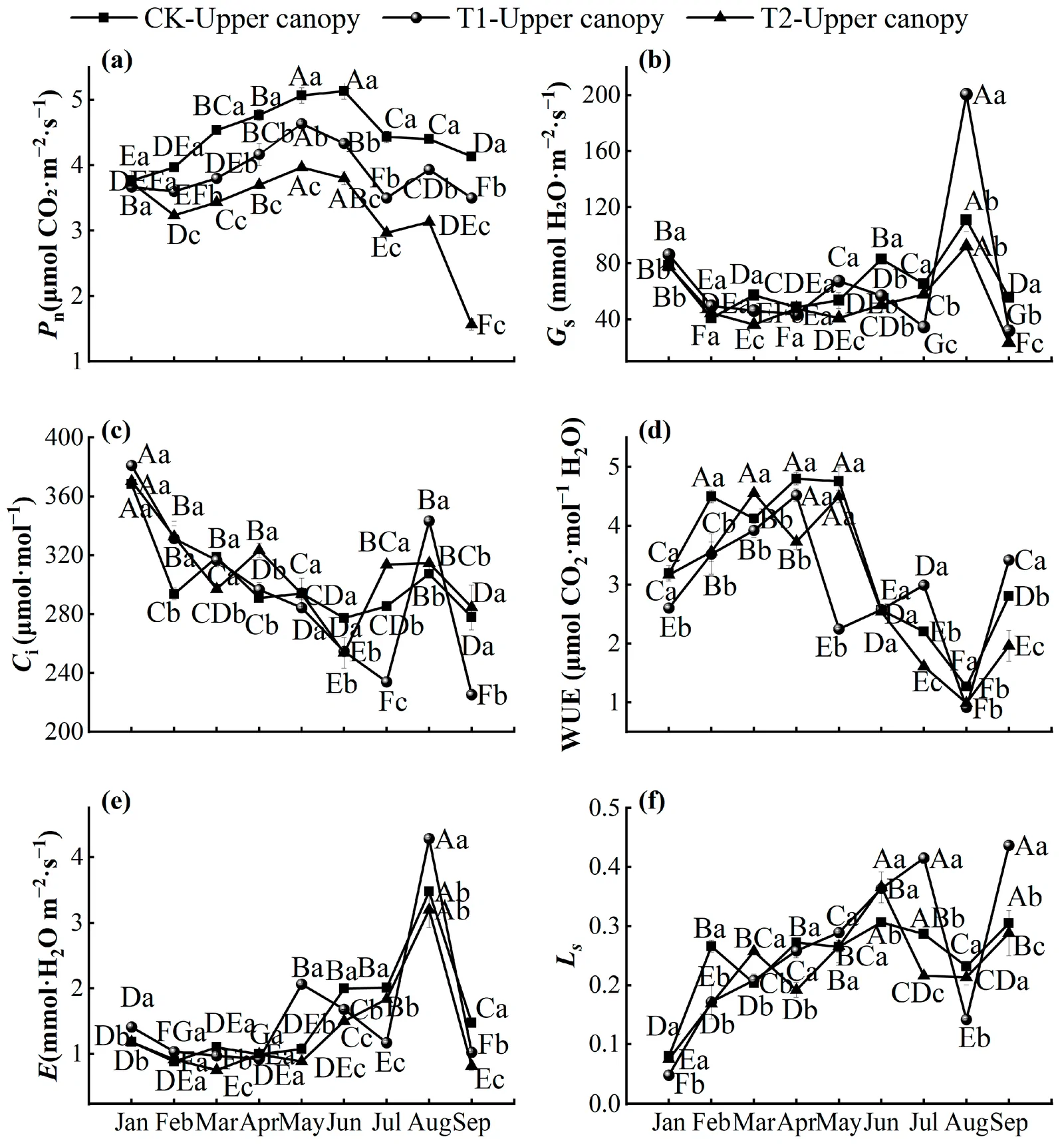
Monthly dynamics of photosynthetic parameters in unshaded upper-canopy needles of Chinese fir. (**a**) Net photosynthetic rate (*P*_n_); (**b**) Stomatal conductance (*G*_s_); (**c**) Intercellular CO_2_ concentration (*C*_i_); (**d**) Water use efficiency (WUE); (**e**) Transpiration rate (*E*); (**f**) Stomatal limitation (*L*_s_). CK: control; T1: moderate shading; T2: severe shading. Note: Different uppercase letters indicate significant differences among months within the same treatment (*p* < 0.05); different lowercase letters indicate significant differences among treatments within the same month (*p* < 0.05). Error bars represent standard error.

**Figure 6:**
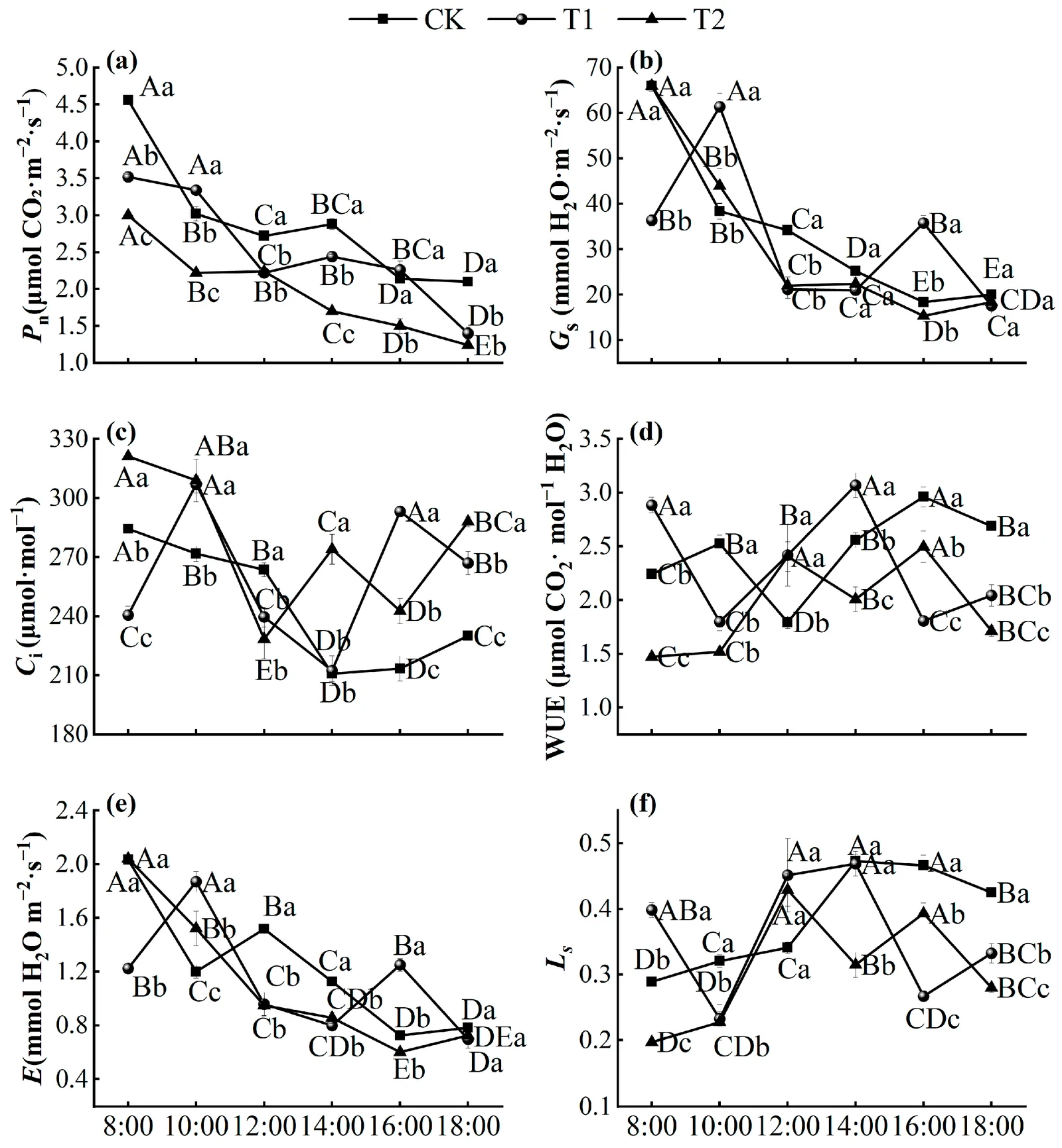
Diurnal dynamics of photosynthetic parameters in upper non-shaded needles of Chinese fir. (**a**) Net photosynthetic rate (*P*_n_); (**b**) Stomatal conductance (*G*_s_); (**c**) Intercellular CO_2_ concentration (*C*_i_); (**d**) Water use efficiency (WUE); (**e**) Transpiration rate (*E*); (**f**) Stomatal limitation (*L*_s_). CK: control; T1: moderate shading; T2: severe shading. Note: Different uppercase letters indicate significant differences among time points within the same treatment (*p* < 0.05); different lowercase letters indicate significant differences among treatments within the same time point (*p* < 0.05). Error bars represent standard error.

**Figure 7:**
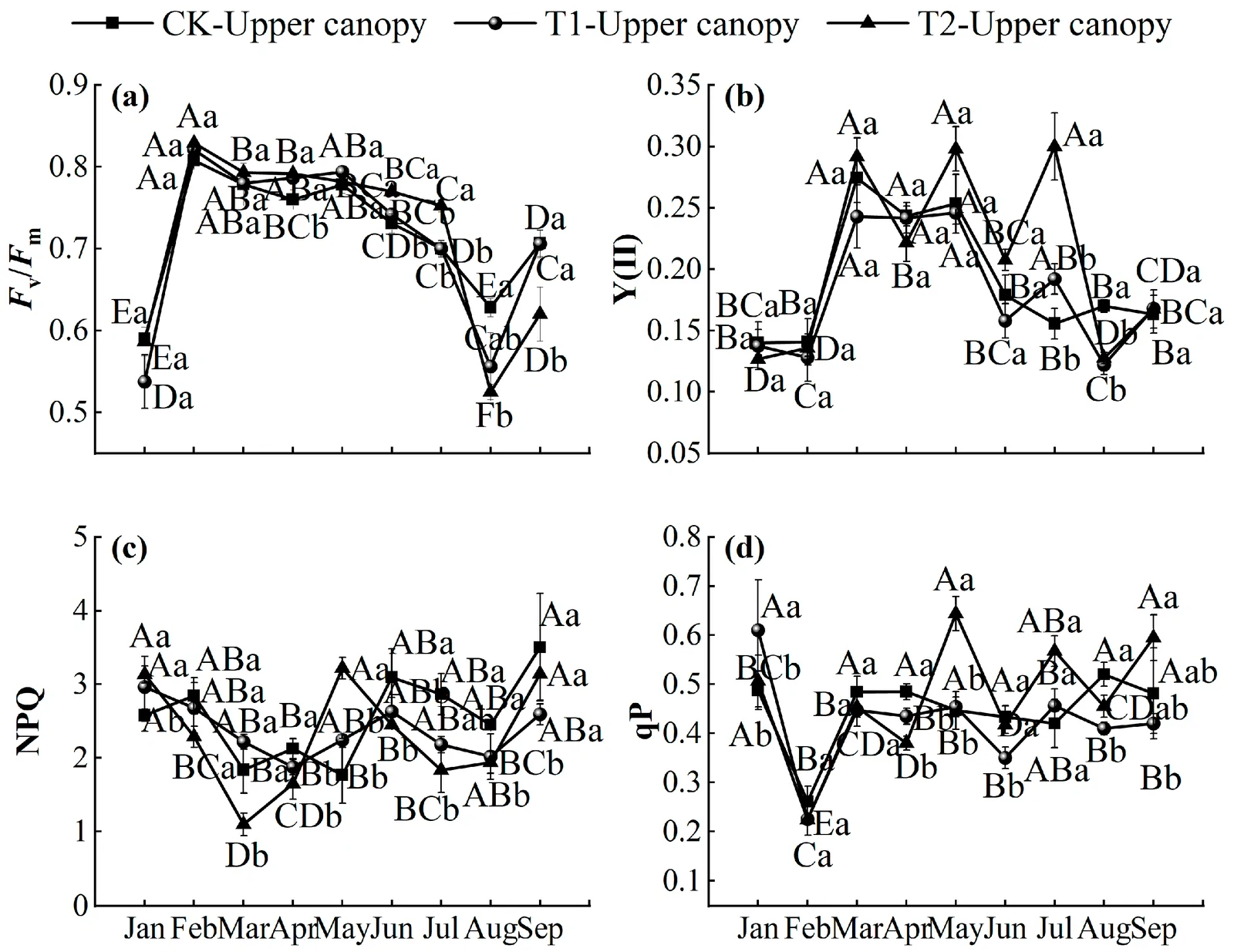
Monthly dynamics of key chlorophyll fluorescence parameters in unshaded upper-canopy needles of Chinese fir. (**a**) Maximum photochemical efficiency (*F*_v_/*F*_m_); (**b**) Actual photochemical efficiency of PSII (Y(II)); (**c**) Non-photochemical quenching (NPQ); (**d**) Photochemical quenching (qP). CK: control; T1: moderate shading; T2: severe shading. Note: Different uppercase letters indicate significant differences among months within the same treatment (*p* < 0.05); different lowercase letters indicate significant differences among treatments in the same month (*p* < 0.05). Error bars represent standard error. The complete dataset is provided in Figure S2.

**Figure 8:**
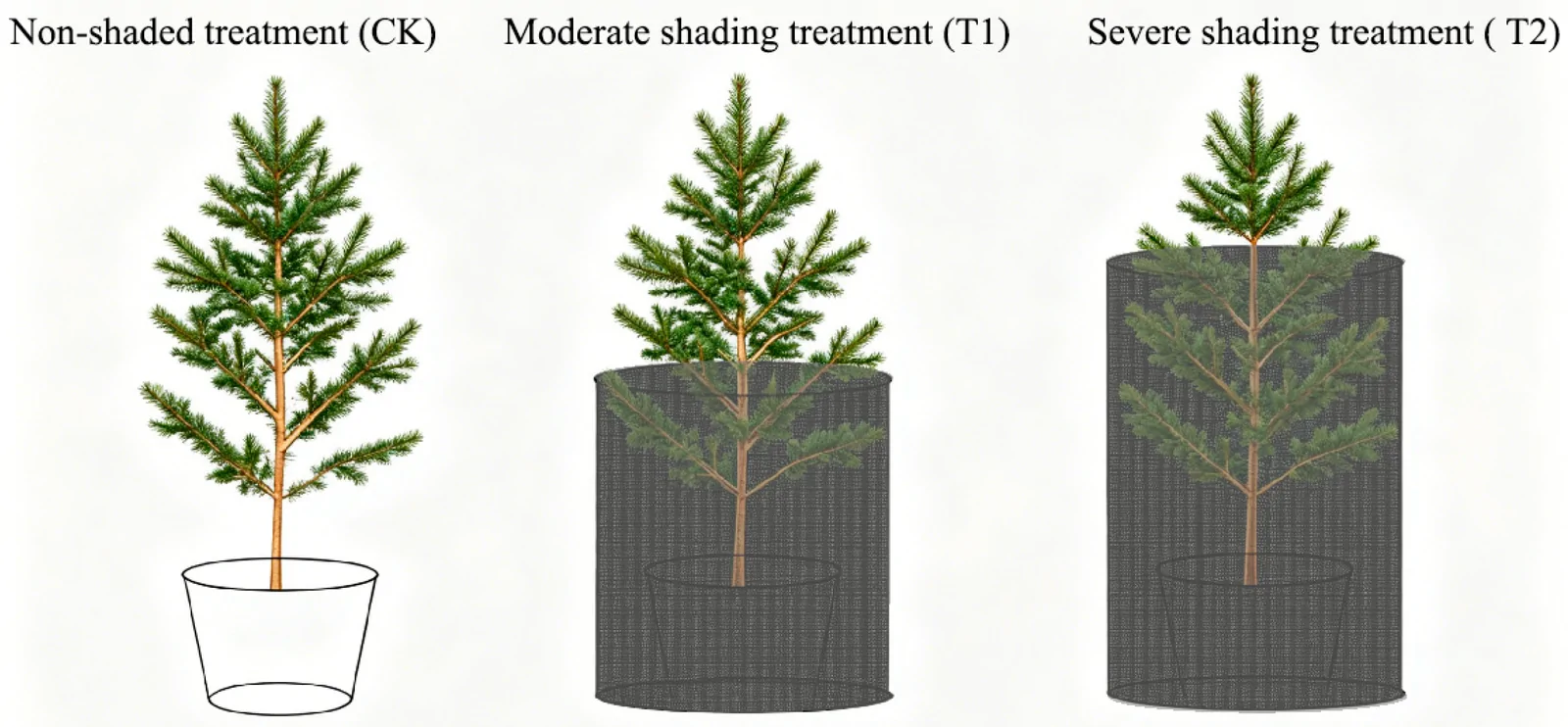
Schematic illustration of gradient shading experiment with potted *C. lanceolata* seedlings.

**Table 1:** Pearson correlation coefficients (*r*) of photosynthetic parameters between lower- and upper-canopy needles across different shading treatments (January–June).

Photosynthetic Parameters	CK		T1		T2	
	*r*	*p*	*r*	*p*	*r*	*p*
Lower canopy *P*_n_ ↔ Upper canopy *P*_n_	0.615 **	0.007	−0.603 **	0.008	−0.356	0.147
Lower canopy *G*_s_ ↔ Upper canopy *G*_s_	0.898 **	<0.01	0.498 *	0.035	0.511 *	0.03
Lower canopy *C*_i_ ↔ Upper canopy *C*_i_	0.736 **	<0.01	0.253	0.311	−0.132	0.6
Lower canopy *L*_s_ ↔ Upper canopy *L*_s_	0.736 **	<0.01	0.253	0.311	−0.132	0.6
Lower canopy *E* ↔ Upper canopy *E*	0.978 **	<0.01	0.084	0.741	−0.213	0.396
Lower canopy WUE ↔ Upper canopy WUE	0.978 **	<0.01	0.606 **	0.008	0.093	0.712
Lower canopy *P*_n_ ↔ Upper canopy *L*_s_	0.812 **	<0.01	−0.842 **	<0.01	−0.897 **	<0.01
Lower canopy *P*_n_ ↔ Upper canopy *C*_i_	−0.812 **	<0.01	0.842 **	<0.01	0.897 **	<0.01

Note: CK: control; T1: moderate shading; T2: severe shading. For each Pearson correlation, 18 paired observations were included per treatment (*n* = 18, df = 16). * *p* < 0.05, ** *p* < 0.01.

**Table 2:** Pearson correlation coefficients (*r*) of chlorophyll fluorescence parameters between lower- and upper-canopy needles across different shading treatments (January–June).

Chlorophyll Fluorescence Parameters	CK		T1		T2	
	*r*	*p*	*r*	*p*	*r*	*p*
Lower canopy *F*_v_/*F*_m_ ↔ Upper canopy *F*_v_/*F*_m_	0.713 **	<0.01	0.792 **	<0.01	0.638 **	<0.01
Lower canopy Y(II) ↔ Upper canopy Y(II)	0.343 *	0.018	0.527 **	0.001	0.546 **	0.001
Lower canopy qP ↔ Upper canopy NPQ	−0.159	0.287	−0.2	0.223	−0.166	0.333
Lower canopy NPQ ↔ Upper canopy qP	−0.004	0.977	0.212	0.196	−0.067	0.696
Lower canopy qP ↔ Upper canopy Y(II)	0.23	0.121	0.334 *	0.038	0.394 *	0.017
Lower canopy *F*_v_/*F*_m_ ↔ Upper canopy qP	−0.024	0.874	0.02	0.902	−0.361 *	0.031
Lower canopy *F*_v_/*F*_m_ ↔ Upper canopy NPQ	0.079	0.598	0.154	0.35	−0.355 *	0.033

Note: CK: control; T1: moderate shading; T2: severe shading. * *p* < 0.05, ** *p* < 0.01.

**Table 3:** Time-lag correlation coefficients (*r*) between senescence stage of lower needles (May–June) and response stage of upper needles (June–September) under different treatments.

Photosynthesis and Fluorescence Parameters	T1		T2	
	*r*	*p*	*r*	*p*
Lower canopy *F*_v_/*F*_m_ ↔ Upper canopy Y(II)	−0.504 *	0.023	−0.472	0.065
Lower canopy NPQ ↔ Upper canopy qP	−0.282	0.229	−0.658 **	0.006
Lower canopy *F*_v_/*F*_m_ ↔ Upper canopy qP	−0.391	0.088	−0.629 **	0.009
Lower canopy *G*_s_ ↔ Upper canopy *G*_s_	0.685 *	0.014	0.586 *	0.045
Lower canopy *P*_n_ ↔ Upper canopy *G*_s_	0.680 *	0.015	0.594 *	0.042
Lower canopy *P*_n_ ↔ Upper canopy *P*_n_	0.893 **	<0.01	0.727 **	0.007
Lower canopy *P*_n_ ↔ Upper canopy *E*	0.711 **	<0.01	0.578 *	0.049

Note: T1: moderate shading; T2: severe shading. * *p* < 0.05, ** *p* < 0.01.

**Table 4:** Basic physicochemical properties of soil in the pot experiment (mean ± SE, *n* = 3).

pH	SWC (%)	BD (g·cm^−3^)	TC (g·kg^−1^)	SOC (g·kg^−1^)	TN (g·kg^−1^)	TP (g·kg^−1^)	TK (g·kg^−1^)
4.69 ± 0.01	19.68 ± 0.83	1.13 ± 0.02	4.00 ± 0.17	2.43 ± 0.15	0.20 ± 0.00	0.18 ± 0.00	1.91 ± 0.02

Note: SWC, soil water content; BD, bulk density; TC, total carbon; SOC, soil organic carbon; TN, total nitrogen; TP, total phosphorus; TK, total potassium.

## Data Availability

All analysed data are presented in the main text and Supplementary Materials.

## Supplementary Materials

The following supporting information can be downloaded at: https://www.mdpi.com/article/10.3390/plants15192948/s1, Table S1: Two-way ANOVA (*F*-values) for the effects of shading treatment, measurement month, and their interaction on photosynthetic parameters of upper and lower canopy needles of *Cunninghamia lanceolata*; Table S2: Two-way ANOVA (*F*-values) for the effects of shading treatment, diurnal time, and their interaction on photosynthetic parameters of upper canopy needles of *Cunninghamia lanceolata*; Table S3: Two-way ANOVA (*F*-values) for the effects of shading treatment, measurement month, and their interaction on chlorophyll fluorescence parameters of upper and lower canopy needles of *Cunninghamia lanceolata*; Figure S1: Monthly dynamics of chlorophyll fluorescence parameters in shaded lower-canopy needles of *Cunninghamia lanceolata*. (a) Minimal fluorescence (*F*_o_); (b) Variable fluorescence (*F*_v_); (c) Maximum fluorescence (*F*_m_); (d) Steady-state fluorescence (*F*_s_); (e) Light-adapted maximum fluorescence (*F*_m_′); (f) Light-adapted minimal fluorescence (*F*_o_′, Cacu); (g) Efficiency of excitation energy capture by open PSII reaction centers (*F*_v_′/*F*_m_′); (h) Relative electron transport rate (rETR(II)); (i) Non-photochemical quenching coefficient (qN); (j) Quantum yield of non-photochemical energy loss in PSII (Y(NO)); (k) Quantum yield of regulated non-photochemical energy dissipation (Y(NPQ)). Different uppercase letters indicate significant differences among months within the same treatment (*p* < 0.05); different lowercase letters indicate significant differences among treatments in the same month (*p* < 0.05). Error bars represent standard error; Figure S2: Monthly dynamics of chlorophyll fluorescence parameters in non-shaded upper-canopy needles of *Cunninghamia lanceolata*. (a) Minimal fluorescence (*F*_o_); (b) Variable fluorescence (*F*_v_); (c) Maximum fluorescence (*F*_m_); (d) Steady-state fluorescence (*F*_s_); (e) Light-adapted maximum fluorescence (*F*_m_′); (f) Light-adapted minimal fluorescence (*F*_o_′, Cacu); (g) Efficiency of excitation energy capture by open PSII reaction centers (*F*_v_′/*F*_m_′); (h) Relative electron transport rate (rETR(II)); (i) Non-photochemical quenching coefficient (qN); (j) Quantum yield of non-photochemical energy loss in PSII (Y(NO)); (k) Quantum yield of regulated non-photochemical energy dissipation (Y(NPQ)). Different uppercase letters indicate significant differences among months within the same treatment (*p* < 0.05); different lowercase letters indicate significant differences among treatments in the same month (*p* < 0.05). Error bars represent standard error.
