# Supplementary material for: Effects of Shading-Induced Senescence and Retention of Lower-Canopy Needles on Photosynthetic Dynamics in Upper-Canopy Needles of Cunninghamia lanceolata
Source: Plants (Basel). 2026 Sep 27;15(19):2948. doi: 10.3390/plants15192948 (PMC13667245; doi:10.3390/plants15192948)
Supplement: Supplementary file 1 [file plants-15-02948-s001.zip › plants-4530386-supplementary.pdf]

Supplementary Materials

Effects of Shading-Induced Senescence and Retention of Lower-Canopy Needles on Photosynthetic Dynamics in Upper-Canopy Needles of *Cunninghamia lanceolata*

Yueyue He[1,2], Yu Zhang[1,2], Qi Liu[1,2,3], Zhihao Li[3,4], Mengyuan Mao[3,4], Shubin Li[1,2] and Lili Zhou[3*]

[1]*College of Forestry, Fujian Agriculture and Forestry University, Fuzhou 350002, China; heeyy0210@126.com (Y.H.); 17805926186@163.com (Y.Z.); lllys021@126.com (Q.L.); fjlishubin@126.com (S.L.)*

[2]*Engineering Research Center of Chinese Fir, National Forestry and Grassland Administration, Fuzhou 350002, China*

[3]*College of Geography and Oceanography, Minjiang University, Fuzhou 350108, China; lzh880881@126.com (Z.L.); maooomy@163.com (M.M.); fjzhoulili@126.com (L.Z.)*

[4]*College of Resources and Environment, Fujian Agriculture and Forestry University, Fuzhou 350002, China*

* *Correspondence: fjzhoulili@126.com*

Number of pages: **6** Number of tables: **3**, Number of pictures: **2**

Table S1 Two-way ANOVA (*F*-values) for the effects of shading treatment, measurement month, and their interaction on photosynthetic parameters of upper and lower canopy needles of *Cunninghamia lanceolata*

Canopy position	Photosynthetic parameters	Shading treatment		Month		Shading × Month	
		F	*P*	*F*	*P*	*F*	*P*
Upper canopy (non-shaded)	P_n	468.68	<0.01	93.94	<0.01	20.01	<0.01
	G_s	82.69	<0.01	325.86	<0.01	58.15	<0.01
	C_i	12.46	<0.01	119.09	<0.01	15.75	<0.01
	WUE	31.48	<0.01	232.38	<0.01	27.96	<0.01
	E	50.14	<0.01	554.17	<0.01	29.94	<0.01
	L_s	12.46	<0.01	119.09	<0.01	15.75	<0.01
Lower canopy (shaded)	P_n	1199.20	<0.01	247.13	<0.01	138.68	<0.01
	G_s	781.55	<0.01	761.20	<0.01	855.69	<0.01
	C_i	107.40	<0.01	184.65	<0.01	42.99	<0.01
	WUE	150.75	<0.01	392.80	<0.01	47.92	<0.01
	E	1306.89	<0.01	1064.12	<0.01	1580.73	<0.01
	L_s	107.40	<0.01	184.65	<0.01	42.99	<0.01

Note: P_n, net photosynthetic rate; G_s, stomatal conductance; C_i, intercellular CO_2 concentration; WUE, water use efficiency; E, transpiration rate; L_s, stomatal limitation.

Table S2 Two-way ANOVA (*F*-values) for the effects of shading treatment, diurnal time, and their interaction on photosynthetic parameters of upper canopy needles of *Cunninghamia lanceolata*

Photosynthetic parameters	Shading treatment		diurnal time		Shading × diurnal time	
	F	*P*	*F*	*P*	*F*	*P*
P_n	23.262	<0.01	97.505	<0.01	11.612	<0.01
G_s	0.898	0.412	59.057	<0.01	9.273	<0.01
C_i	0.53	0.591	14.358	<0.01	8.585	<0.01
WUE	1.196	0.308	4.288	0.002	10.632	<0.01
E	1.315	0.275	38.535	<0.01	7.786	<0.01
L_s	0.53	0.591	14.358	<0.01	8.585	<0.01

Note: P_n, net photosynthetic rate; G_s, stomatal conductance; C_i, intercellular CO_2 concentration; WUE, water use efficiency; E, transpiration rate; L_s, stomatal limitation.

Table S3 Two-way ANOVA (F-values) for the effects of shading treatment, measurement month, and their interaction on Chlorophyll fluorescence parameters of upper and lower canopy needles of *Cunninghamia lanceolata*

Canopy position	Chlorophyll fluorescence parameters	Shading treatment		Month		Shading × Month	
		F	P	F	P	F	P
Upper canopy (non-shaded)	F_v/F_m	0.40	0.669	99.42	<0.01	3.51	<0.01
	Y(II)	4.32	0.015	29.89	<0.01	2.48	0.002
	NPQ	1.33	0.268	5.07	<0.01	1.59	0.078
	qP	2.37	0.097	19.09	<0.01	2.00	0.017
	F_o	3.32	0.039	31.17	<0.01	2.39	0.003
	F_v	1.27	0.285	13.69	<0.01	0.58	0.892
	F_m	1.85	0.161	4.72	<0.01	0.57	0.9
	F_s	2.57	0.08	5.87	<0.01	3.19	<0.01
	F_m'	3.13	0.047	12.34	<0.01	3.86	<0.01
	F_o'(Cacu)	3.81	0.024	7.68	<0.01	2.34	0.004
	F_v'/F_m'	1.60	0.206	67.19	<0.01	4.01	<0.01
	rETR(II)	4.23	0.016	30.03	<0.01	2.51	0.002
	qN	1.72	0.182	11.15	<0.01	3.18	<0.01
	Y(NO)	0.42	0.658	4.56	<0.01	2.19	0.008
	Y(NPQ)	2.98	0.054	17.64	<0.01	2.31	0.005
Lower canopy (shaded)	F_v/F_m	19.34	<0.01	99.92	<0.01	13.28	<0.01
	Y(II)	4.04	0.021	57.63	<0.01	1.90	0.053
	NPQ	4.64	0.012	13.98	<0.01	2.44	0.012
	qP	1.04	0.357	64.67	<0.01	2.33	0.016
	F_o	11.23	<0.01	49.56	<0.01	2.60	0.007
	F_v	8.31	<0.01	29.48	<0.01	7.83	<0.01
	F_m	7.98	0.001	17.30	<0.01	6.34	<0.01
	F_s	4.42	0.014	12.95	<0.01	2.44	0.012
	F_m'	4.80	0.01	6.95	<0.01	3.02	0.002
	Fo'(Cacu)	10.69	<0.01	8.11	<0.01	1.62	0.112
	F_v'/F_m'	1.37	0.259	48.40	<0.01	4.48	<0.01
	rETR(II)	2.43	0.093	42.58	<0.01	1.23	0.285
	qN	5.66	0.005	15.14	<0.01	3.16	0.001
	Y(NO)	7.68	0.001	9.70	<0.01	2.15	0.027
	Y(NPQ)	4.63	0.012	40.55	<0.01	4.24	<0.01

Note: F_v/F_m, maximum photochemical efficiency; Y(II), actual photochemical efficiency of PSII; NPQ, non-photochemical quenching; qP, photochemical quenching; F_o, minimal fluorescence; F_v, variable fluorescence; F_m, maximum fluorescence; F_s, steady-state fluorescence; F_m', light-adapted maximum fluorescence; Fo'(Cacu), light-adapted minimal fluorescence; F_v'/F_m', efficiency of excitation energy capture by open PSII reaction centers; rETR(II), relative electron transport rate; qN, non-photochemical quenching coefficient; Y(NO), quantum yield of non-regulated non-photochemical energy loss in PSII; Y(NPQ), quantum yield of regulated non-photochemical energy dissipation.

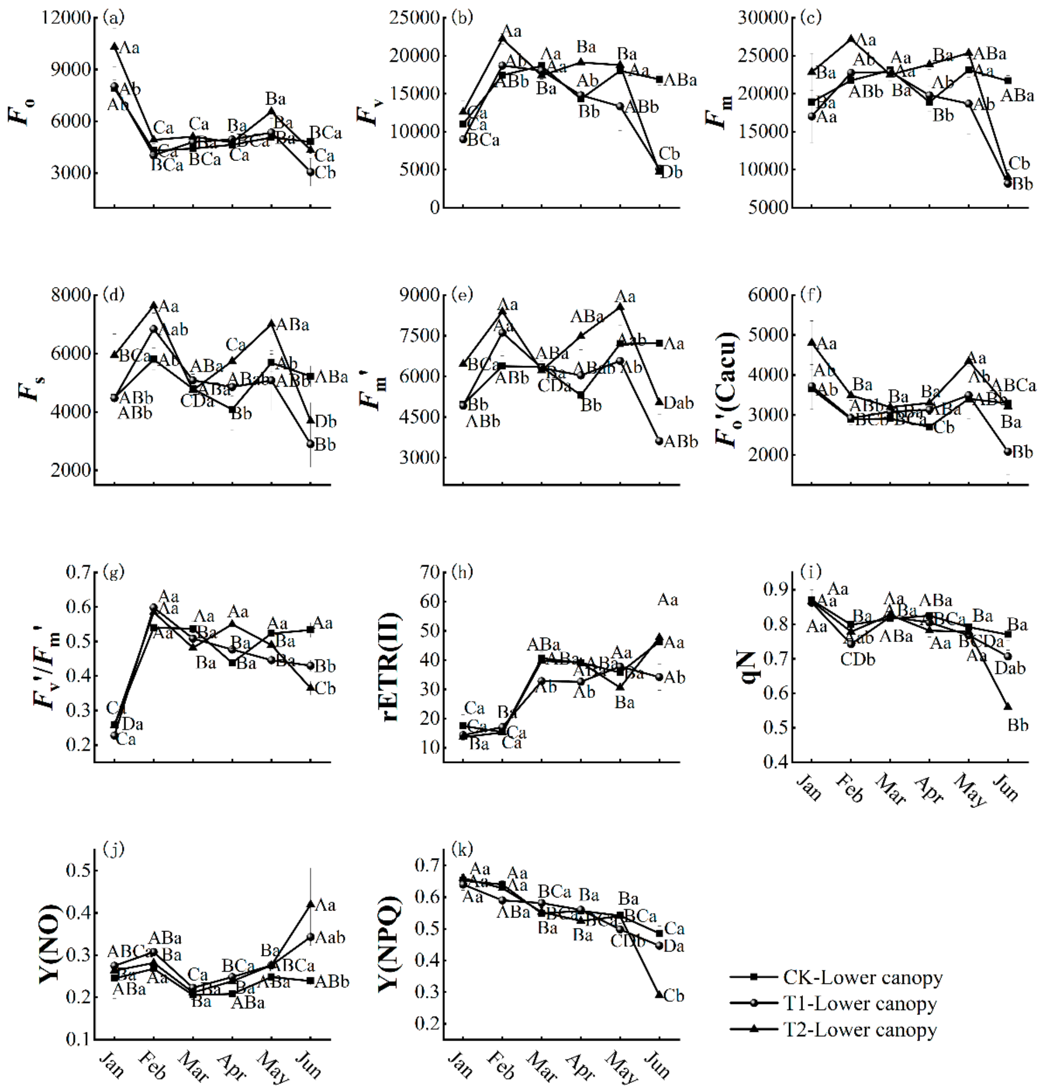

Figure S1 Monthly dynamics of chlorophyll fluorescence parameters in shaded lower-canopy needles of *Cunninghamia lanceolata*. (a) Minimal fluorescence (F_o); (b) Variable fluorescence (F_v); (c) Maximum fluorescence (F_m); (d) Steady-state fluorescence (F_s); (e) Light-adapted maximum fluorescence (F_m'); (f) Light-adapted minimal fluorescence (F_o', Cacu); (g) Efficiency of excitation energy capture by open PSII reaction centers (F_v'/F_m'); (h) Relative electron transport rate (rETR(II)); (i) Non-photochemical quenching coefficient (qN); (j) Quantum yield of non-photochemical energy loss in PSII (Y(NO)); (k) Quantum yield of regulated non-photochemical energy dissipation (Y(NPQ)). Different uppercase letters indicate significant differences among months within the same treatment ($p < 0.05$); different lowercase letters indicate significant differences among treatments in the same month ($p < 0.05$). Error bars represent standard error.

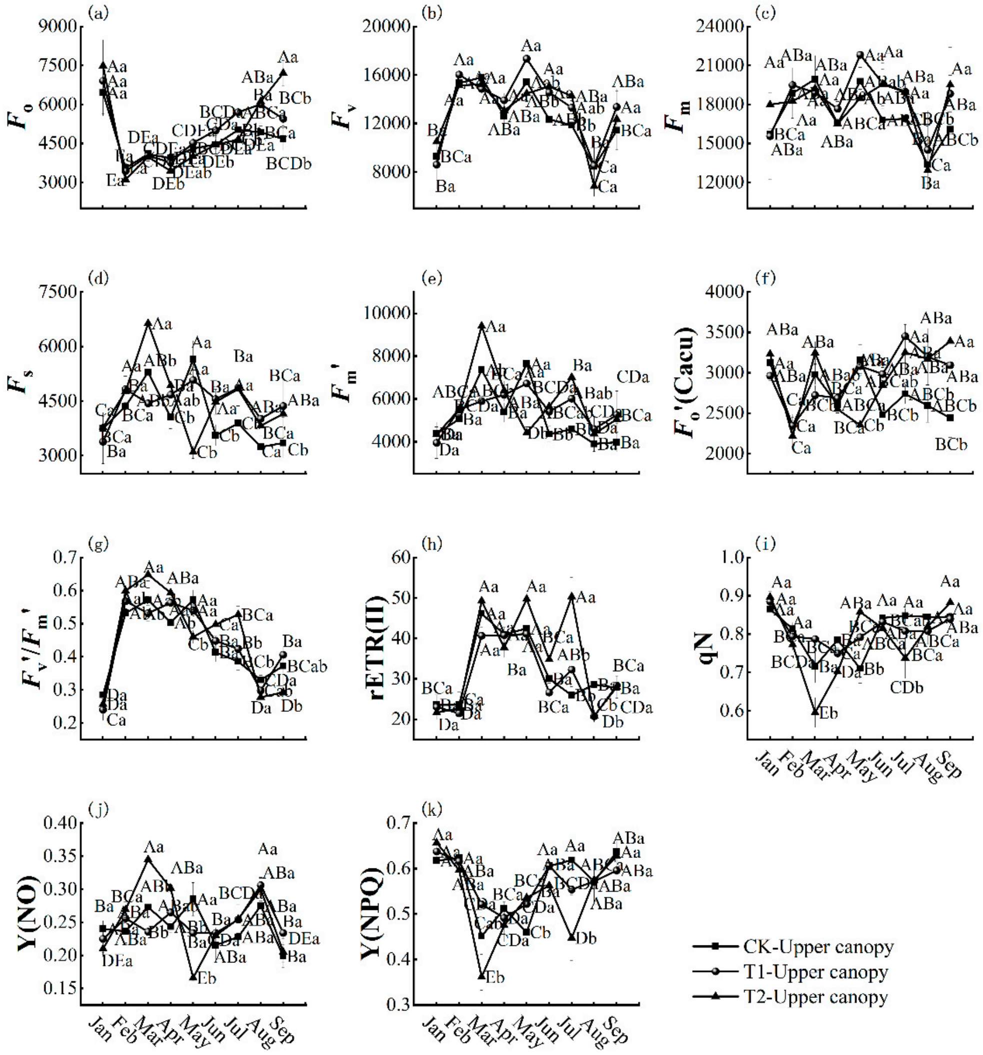

Figure S2 Monthly dynamics of chlorophyll fluorescence parameters in non-shaded upper-canopy needles of *Cunninghamia lanceolata*. (a) Minimal fluorescence (F_o); (b) Variable fluorescence (F_v); (c) Maximum fluorescence (F_m); (d) Steady-state fluorescence (F_s); (e) Light-adapted maximum fluorescence (F_m'); (f) Light-adapted minimal fluorescence (F_o', Cacu); (g) Efficiency of excitation energy capture by open PSII reaction centers (F_v'/F_m'); (h) Relative electron transport rate (rETR(II)); (i) Non-photochemical quenching coefficient (qN); (j) Quantum yield of non-photochemical energy loss in PSII (Y(NO)); (k) Quantum yield of regulated non-photochemical energy dissipation (Y(NPQ)). Different uppercase letters indicate significant differences among months within the same treatment ($p < 0.05$); different lowercase letters indicate significant differences among treatments in the same month ($p < 0.05$). Error bars represent standard error.
